# Draft Genome Sequence and Annotation of the Halotolerant Carotenoid-Producing Strain Paracoccus bogoriensis BOG6^T^

**DOI:** 10.1128/mra.00133-23

**Published:** 2023-04-17

**Authors:** Upasana Pal, Denise Bachmann, Lars M. Blank, Till Tiso

**Affiliations:** a Institute of Applied Microbiology, RWTH Aachen University, Aachen, Germany; Portland State University

## Abstract

*Paracoccus* spp. are Gram-negative, coccoid bacteria, fascinating for their ability to grow in highly diverse environments while producing commercially relevant products. This study describes the draft genome sequence of the halotolerant, alkaliphilic, and thermotolerant carotenoid-producing type strain Paracoccus bogoriensis BOG6^T^.

## ANNOUNCEMENT

Paracoccus bogoriensis BOG6^T^ (DSM 16578) was isolated from Lake Bogoria, a saline, alkaline lake located in the Kenyan Rift Valley. The lake waters feature pH values ranging from 9 to 11 (1, 2) and dissolved salt concentrations of >40% (2). The lake’s temperature varies between 30°C and 90°C. *P. bogoriensis* BOG6^T^ is motile, possessing a single flagellum (1). The strain is capable of accumulating polyhydroxybutyrate granules (1). Strain BOG6^T^ produces the industrially interesting carotenoid astaxanthin, with a yield of 0.4 mg/g of wet cells (1).

Strain DSM 16578^T^ was purchased from the German Collection of Microorganisms and Cell Cultures (DSMZ, Braunschweig, Germany). It was grown aerobically in DSMZ medium 1325 (pH 9.5) (3) at 30°C. Genomic DNA was harvested during the exponential phase and extracted using the Monarch genomic DNA purification kit (New England Biolabs, Frankfurt, Germany). To further enhance the quality of the genomic DNA, it was purified using the Monarch PCR and DNA cleanup kit (New England Biolabs). Sequencing of the whole genome of *P. bogoriensis* BOG6^T^ was performed using the Illumina NextSeq instrument (4), followed by *de novo* assembly. A library was prepared using Illumina genome sequencing technology on the NovaSeq 6000 S4 XP instrument in paired-end, 150-bp (PE150) sequencing mode (Eurofins Genomics, Ebersberg, Germany). To enhance the quality of the sequence, Trimmomatic version 0.33 was used for sequence cleaning prior to assembly (5). Finally, *de novo* genome assembly was performed using SPAdes version 3.10.1 (3, 6). The assembly was further evaluated using QUAST (Quality Assessment Tool for Genome Assemblies) version 5.0.2 software (7). Default parameters were used for the above software.

The sequencing run yielded a total of 10,843,158 bp paired-end 2 × 150-bp reads. The assembly consisted of 97 contigs with an *N*_50_ value of 105,619 bp. The total genome size was 3,012,291 bp, and the total GC content was 66.6% (8). Furthermore, the QUAST output provided a genome mapping of 99.5%. The sequence was submitted to NCBI, and annotation was performed using the NCBI Prokaryotic Genome Annotation Pipeline (PGAP) (9, 10). The genome of *Paracoccus bogoriensis* BOG6^T^ comprises a total of 2,922 protein coding sequences, 48 noncoding RNAs, including 1 each of 16S, 23S, and 5S rRNA genes, and 42 tRNA genes. Finally, the *Paracoccus bogoriensis* BOG6^T^ genome sequence provides insightful information about the presence of genes facilitating halotolerance, including the Trk system with the potassium uptake protein TrkA (11), which aids in survival under high-salinity conditions. Another interesting trait is the presence of gene *crtY*, coding for the enzyme lycopene β-cyclase (12), responsible for the synthesis of an industrially relevant β-carotene (13).

### Data availability.

The whole-genome shotgun sequence, including the metadata, has been deposited at DDBJ/ENA/GenBank under the accession number JAHYSR000000000. The version described in this paper is version JAHYSR000000000.1 and consists of JAHYSR010000001 to JAHYSR010000097. The raw sequencing reads have been deposited in the SRA and are available under accession number SRR23977133.
